# Reciprocal and unidirectional scattering of parity-time symmetric structures

**DOI:** 10.1038/srep20976

**Published:** 2016-02-15

**Authors:** L. Jin, X. Z. Zhang, G. Zhang, Z. Song

**Affiliations:** 1 Nankai University, School of Physics, Tianjin, 300071, P. R. China; 2 Tianjin Normal University, College of Physics and Materials Science, Tianjin, 300387, P. R. China

## Abstract

Parity-time 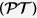 symmetry is of great interest. The reciprocal and unidirectional features are intriguing besides the 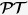 symmetry phase transition. Recently, the reciprocal transmission, unidirectional reflectionless and invisibility are intensively studied. Here, we show the reciprocal reflection/transmission in 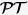-symmetric system is closely related to the type of 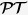 symmetry, that is, the axial (reflection) 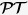 symmetry leads to reciprocal reflection (transmission). The results are further elucidated by studying the scattering of rhombic ring form coupled resonators with enclosed synthetic magnetic flux. The nonreciprocal phase shift induced by the magnetic flux and gain/loss break the parity 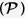 and time-reversal 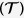 symmetry but keep the parity-time 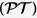 symmetry. The reciprocal reflection (transmission) and unidirectional transmission (reflection) are found in the axial (reflection) 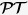-symmetric ring centre. The explorations of symmetry and asymmetry from 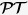 symmetry may shed light on novel one-way optical devices and application of 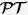-symmetric metamaterials.

Parity-time 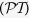 symmetric quantum system may possess entirely real spectrum although being non-Hermitian^1^,^2^,^3^,^4^,^5^,^6^,^7^,^8^,^9^,^10^,^11^,^12^,^13^. 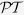 symmetric system is invariant under the combined 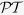 operator in the presence of balanced gain and loss. In the past decade, 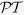-symmetric system has attracted tremendous interests as it possesses unintuitive but intriguing implications. Due to the similarity between the paraxial wave equation describing spatial light wave propagation and the temporal Schrödinger equation for quantum system, the complex refractive index distribution satisfying n^*^(*x*) = *n*(−*x*) mimics 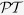-symmetric potentials V^*^(*x*) = *V*(−*x*), 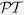-symmetric systems are proposed and realized in coupled optical waveguides through index guiding and a inclusion of balanced gain and loss regions^14^,^15^,^16^,^17^. A number of novel and non-trivial phenomena are found, such as power oscillation^17^, coherent perfect absorbers^18^,^19^,^20^, nonreciprocal light propagation^21^ in coupled waveguides, and recently the 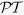-symmetric microcavity lasing^22^,^23^,^24^ and gain induced large optical nonlinear^25^,^26^,^27^,^28^,^29^ in coupled resonators.

The spectral singularity^30^,^31^,^32^,^33^,^34^,^35^,^36^ and invisibility^37^,^38^,^39^,^40^,^41^,^42^,^43^ in 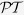-symmetric system are hot topics, where reciprocal transmission and unidirectional reflectionless in 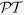-symmetric metamaterial are intriguing features for novel optical devises. These devices are useful for light transport, control and manipulation^44^,^45^,^46^,^47^. The symmetric scattering properties are usually attributed to certain internal symmetry of a scattering centre. For instance, the parity 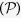 symmetry, or time-reversal 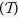 symmetry of a scattering centre leads to symmetric reflection and transmission^48^ (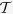-symmetric system without unequal tunnelling amplitude is Hermitian, otherwise, only reciprocal reflection or transmission holds^49^). Here, we report reciprocal reflection, similar as reciprocal transmission, are both related to the 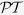 symmetry of a scattering centre: The axial (refection) 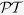 symmetry, with respect to the input and output channels, induces reciprocal reflection (transmission). Recent efforts on photonic Aharonov-Bohm effect enable photons behaving like electrons in magnetic field. Effective magnetic field for photons can be introduced in coupled waveguides by bending the waveguides^50^, periodically modulating the refractive index^51^, and the photon-phonon interactions^52^; or in coupled resonators by magneto-optical effect^53^, dynamic modulation^54^, and off-resonance coupling paths imbalance^55^,^56^. In this work, we focus on the 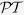-symmetric structure with balanced gain and loss threading by synthetic magnetic flux, where photons feel a nonreciprocal tunnelling phase between neighbour resonators. The nonreciprocal tunnellings and balanced gain and loss break the 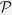 and 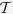 symmetry but keep the 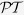 symmetry of the scattering centre. The axial (reflection) 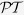 symmetry will lead to reciprocal reflection (transmission) and unidirectional transmission (reflection). Our findings provide new insights of 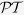 symmetry and the symmetric/asymmetric scattering, which are instrumental for the applications of 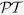-symmetric metamaterial for light transport and one-way optical devises.

## Results

### Reciprocal and unidirectional scattering of 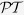 -symmetric structures

**Figure 1:**
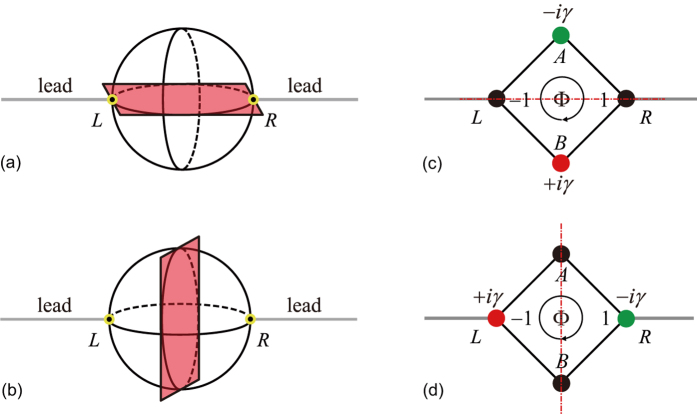
The type of 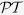 symmetry. Two semi-infinite input and output leads (solid grey) are connected to the 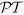-symmetric structures (black sphere) at sites *L* and *R* (yellow circle). (**a**) The axial 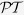 symmetry, defined as self-correspondence of *L*, *R* under parity operation. (**b**) The reflection 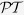 symmetry, defined as reflection-correspondence of *L*, *R* under parity operation. The axial (**c**) and reflection (**d**) 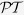-symmetric rhombic ring configurations with enclosed magnetic flux Φ are schematically illustrated. The 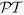 symmetry axes are in dash dotted red. The red (green) site represents the gain (loss).

The symmetric scattering properties of a 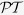-symmetric structure are closely related to the classification of 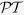-symmetry. The parity operator 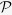 is the spatial reflection operator, 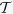 is the time-reversal operator. In Fig. 1a,b, we schematically show two types of 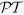 symmetry. The Hamiltonian of the scattering centre is 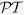-invariant, i.e., 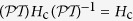. The input and output leads are connected to the 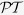-symmetric scattering centre at sites *L* and *R*. If the connection sites under the parity operation satisfies 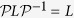, 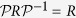, the system is called axial 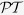 symmetric (Fig. 1a). If the connection sites under the parity operation satisfies 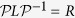, 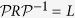, the system is called reflection 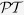 symmetric (Fig. 1b). The red plane indicates the up-to-down (left-to-right) spatial reflection correspondence of axial (reflection) 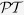 symmetry.

In order to address the reciprocal and unidirectional scattering behavior. We study the reflection and transmission of a scattering centre for the left side and right side inputs, respectively. The Hamiltonian of the scattering system is *H* = *H*_L_ + *H*_c_ + *H*_R_ with *H*_L_ (*H*_R_) being the Hamiltonian of the left (right) lead. We denote the two scattering states as 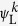 and 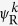 for the input with wave vector *k*. The forward going and backward going waves are in form of 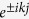. Combining with the reflection and transmission coefficient, we assume the scattering state wave function on the leads (not at the spectral singularities, see Methods) of left side input as,


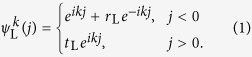


where *r*_L_ and *t*_L_ represent the reflection and transmission coefficients for the left side input with wave vector *k*. Similarly, the wave function on the leads of right side input is in form of


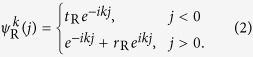


where *r*_R_ and *t*_R_ represent the reflection and transmission coefficients for the right side input with wave vector *k*.

### Reciprocal reflection under axial 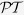 symmetry

As shown in Fig. 1a, this type of 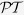-symmetric scattering centres have connection sites under parity operation satisfying 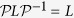, 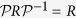. In the axial 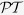-symmetric configuration, the 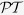 symmetry is defined as 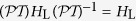, 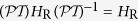 in the leads, and as 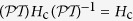 in the centre. The whole scattering system is axial 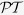-symmetric with respect to the leads. The axial 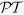 symmetry results in symmetric relations on the scattering coefficients as (see Supplementary Information Note 1 for details),


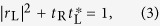



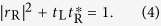


From equations (3,4), we notice the reflection probabilities for the left and right side inputs are the same, i.e.,


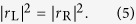


In other words, the axial 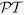 symmetry leads to the reciprocal reflection. Notice that we have reciprocal reflection 
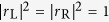
 at any transmission zero *t*_L,R_ = 0, where one-way pass through is possible. Furthermore,considering the waves with vectors *k* and −*k*, the reflection and transmission coefficients further satisfy 
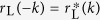
, 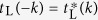, 
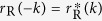
, and 
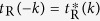
.

### Reciprocal transmission under reflection 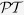 symmetry

As shown in Fig. 1b, this type of 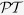-symmetric scattering centres have connection sites under parity operation satisfying 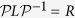, 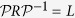. In the reflection 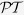-symmetric configuration, the 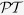 symmetry is defined as 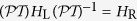, 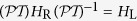 in the leads, and as 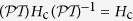 in the centre. The whole scattering system is reflection 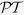-symmetric with respect to the leads. The reflection 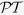 symmetry results in symmetric relations on the scattering coefficients as (see Supplementary Information Note 2 for details),


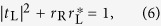



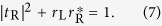


From equations (6,7), we notice the transmission probabilities for the left side and right side inputs are the same, i.e.,


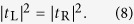


This indicates the reflection 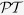 symmetry leads to the reciprocal transmission, as observed in Bragg gratings and other 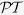-symmetric structures^45^,^46^,^47^. Notice that we have reciprocal transmission 
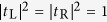
 at any reflection zero *r*_L,R_ = 0, where unidirectional reflectionless is possible^37^,^38^,^39^,^40^,^41^,^42^,^43^. Furthermore, considering the waves with vectors *k* and −*k*, the reflection and transmission coefficients further satisfy 
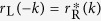
, 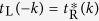, 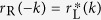, and 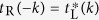.

We show that in the present of 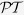 symmetry, the reciprocal reflection or transmission in a scattering centre is protected when the axial or reflection 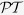 symmetry holds even though the 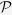 and 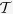 symmetry are absent. Moreover, 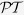 symmetry structure may exhibit unidirectional scattering behavior.

### 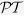 -symmetric rhombic ring structures

We use a rhombic ring structure (Fig. 1c,d) to elucidate the results. The scattering centre encloses with an effective magnetic flux 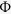, photons moving along the rhombic ring structure in clockwise (counterclockwise) direction will acquire an additional direction-dependent phase factor 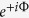 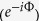, thus photons tunnelling is nonreciprocal except when 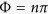, 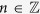. This is an effective photon Aharonov-Bohm effect creating by synthetic magnetic field^50^,^51^,^52^,^53^,^54^,^55^,^56^. The phase factor 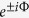 is an analytical function of 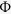 with period of 2*π*, it is sufficient to understand the influence of magnetic flux on the scattering by studying 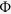 in the region 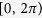. To realize a synthetic magnetic field, two ring resonators are coupled through an auxiliary off-resonant ring resonator. The auxiliary resonator introduces optical paths imbalance when coupling to two primary resonators, the auxiliary resonator can be effectively reduced and create a coupling phase factor between two primary resonators. The coupled resonators under synthesized magnetic field is described by a magnetic tight-binding Hamiltonian^55^,^56^,


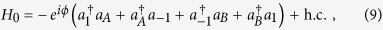


where *ϕ* = Φ/4 is a nonreciprocal phase shift induced by the magnetic flux in the tunnelling constant. In Fig. 1c, the Hamiltonian of the scattering centre is 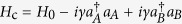, where *γ* is the gain/loss rate. The balanced gain and loss are the origin of the non-Hermiticity realized in the optical systems^14^,^15^,^16^,^17^,^23^,^24^,^25^,^26^,^27^. The configuration is axial 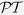-symmetric with the parity operator acting on the rhombic ring sites defined as 
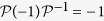
, 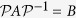, 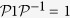, 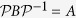. In Fig. 1d, the Hamiltonian of the scattering centre is 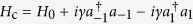, the parity operator 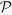 is defined as 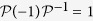, 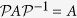, 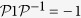, 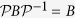, and the configuration is reflection 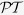-symmetric. In the system, the magnetic flux is inverted meanwhile the gain and loss are switched under the 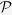 or 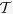 operation. However, the system is invariant under the combined 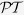 operator, i.e., the presence of non-trivial magnetic flux as well as balanced gain and loss both break the 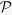 and 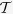 symmetry but keep the 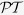 symmetry of the system.

The scattering centre is actually a two-arm Aharonov-Bohm interferometer. Light wave propagates through two pathways (*A* and *B*) between the connection sites −1, 1 and interfere with each other. The interference generates the output which varies as the enclosed magnetic flux. The effective magnetic field is gauge invariant and the magnetic flux acts globally, thus the reflection and transmission are not affected by the nonreciprocal phase distribution in the tunnellings for fixed magnetic flux. In the following, we discuss the reflection and transmission of the 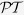-symmetric rhombic ring structures in details.

The reflection and transmission coefficients for the axial 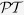-symmetric rhombic ring structure (Fig. 1c) are calculated from the Schrödinger equations (see Methods), yielding


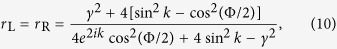



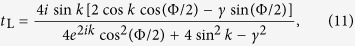



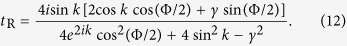


**Figure 2:**
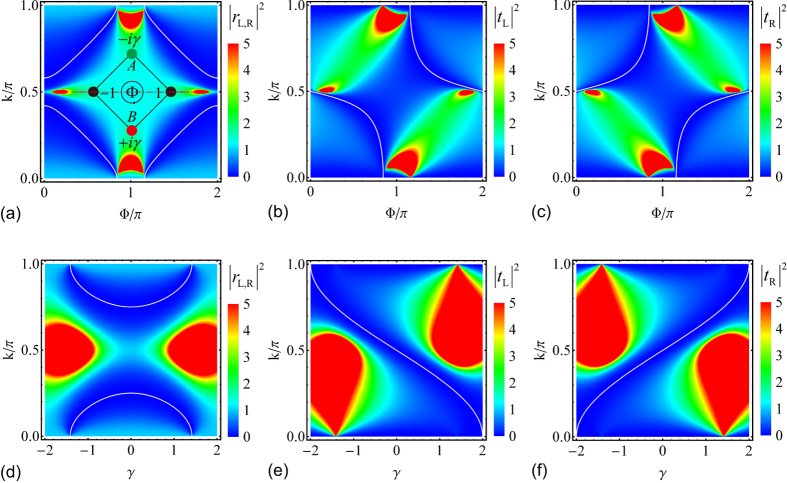
Reciprocal reflection and unidirectional transmission under axial 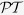 symmetry. (**a**–**c**) The reflection and transmission probabilities 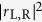, 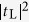, 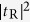 at *γ* = 1/2 as functions of Φ and *k*. (**d**–**f**) The reflection and transmission probabilities 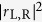, 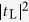, 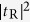 at Φ = *π*/2 as functions of *γ* and *k*. The insert in (**a**) schematically illustrates the axial 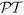-symmetric rhombic ring structure. The white curves are the reflection and transmission zeros. At *k* = *π*/2 and Φ = 0, 2*π*, the reflections in (**a**) are 1, the transmissions in (**b**,**c**) are 0.

The reflection and transmission probabilities are functions of the magnetic flux Φ, gain/loss rate *γ*, and wave vector *k*. They satisfy 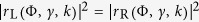 (see Fig. 2a,d), 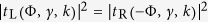 (see Fig. 2b,c), and 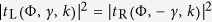 (see Fig. 2e,f). In the absence of non-trivial magnetic flux Φ, or gain/loss *γ*, the system is 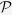-symmetric (reflection-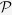-symmetric for Φ absence, i.e., left to right by mirror imaging; inversion-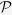-symmetric for *γ* absence, i.e., left to right by 180° rotation), the reflection and transmission are both reciprocal. The non-trivial magnetic flux Φ together with balanced gain and loss *γ* break the 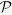 symmetry. The symmetric transmission in the 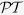-symmetric system at 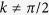 is broken, i.e., the transmission is unidirectional at 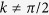. Moreover, the axial 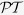 symmetry protects the symmetric reflection, therefore, the reflection is reciprocal but the transmission is unidirectional. The white curves in Fig. 2 show the reflection/transmission zeros. At 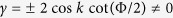, we have *t*_L_ = 0 or *t*_R_ = 0 with total reflection 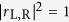. This indicates that we only have a non-zero transmission for the right side or left side input, thus the axial 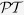-symmetric rhombic ring structure allows one-way pass through.

Photons circle in the scattering centre either in a clockwise direction or in a counterclockwise direction, we schematically illustrate the two pathways in Supplementary Fig. 1. The phase difference between two pathways affects the interference in the scattering centre, thus the transmission varies as the effective magnetic flux induced phase difference. The phase difference between clockwise direction and counterclockwise direction of transmission pathways is Φ for the left side input (Supplementary Fig. 1c) or −Φ for the right side input (Supplementary Fig. 1d). The transmission pathways are not equivalent in the presence of gain/loss, the interference of phase difference being Φ is different from the interference of phase difference being −Φ. Therefore, the unidirectional transmission is enabled in the presence of nonreciprocal tunneling phase factor 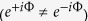 attributed to non-trivial magnetic flux (Φ ≠ *nπ*, 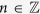).

**Figure 3:**
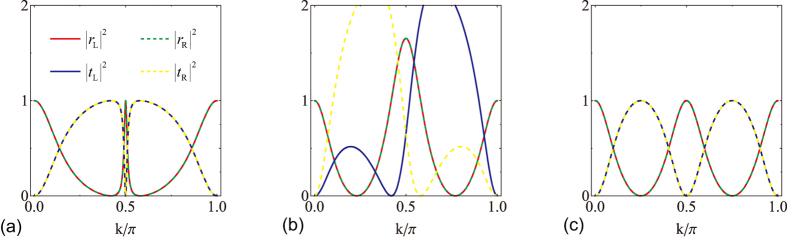
Symmetric reflection under axial 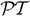 symmetry. (**a**) *γ* = 1/2, Φ = 0, (**b**) *γ* = 1/2, Φ = *π*/2, (**c**) *γ* = 0, Φ = *π*/2.

In Fig. 3, we plot the reflection and transmission probabilities for an axial 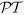-symmetric rhombic ring structure (Fig. 1c) at several set parameters. Figure 3a is for a system with balanced gain and loss in the absence of magnetic flux, i.e., *γ* = 1/2, Φ = 0. The gain/loss, being non-Hermitian, plays the role of on-site potentials and is the origin of unidirectional behavior. However, the presence of balanced gain and loss alone does not ensure unidirectional scattering. We notice the reflection and transmission in Fig. 3a are both reciprocal. The scattering is unitary even though the system is non-Hermitian (the balanced gain and loss of this rhombic ring structure 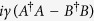 can be reduced to an anti-Hermitian interaction 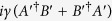 by composing 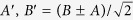, and the non-Hermiticity of the scattering centre only arises from the anti-Hermitian interaction between 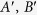, which is proved to have unitary scattering^57^). By introducing magnetic flux to the system, the 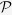 and 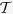 symmetry is destroyed but the 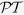 symmetry holds. The interference between light waves from the loss arm and the gain arm generates unidirectional transmission for non-trivial magnetic flux. Figure 3b is for a system in the presence of non-trivial magnetic flux, i.e., *γ* = 1/2, Φ = *π*/2. The unidirectional transmission zero happens at 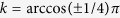, i.e., at *k* ≈ 0.420*π*, 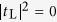, 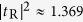; at *k* ≈ 0.580*π*, 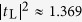, 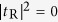, which indicates a one-way pass through. Figure 3c is for a Hermitian scattering centre in the presence of non-trivial magnetic flux, i.e., *γ* = 0, Φ = *π*/2, we have Hermitian scattering without unidirectional behavior.

In the rhombic ring structure under axial 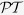 symmetry (Fig. 1c), the reflection and transmission coefficients *r*_L_, *r*_R_, *t*_L_, *t*_R_ diverge at the spectral singularities^30^. When *k* = *π*/2, we have the reflection and transmission coefficients 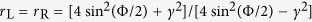 and 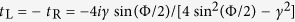. We notice the spectral singularities are at 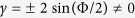. When Φ = *π*, we have the reflection and transmission coefficients 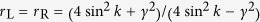 and 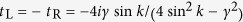. The spectral singularities are at 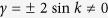. At the spectral singularities, the scattering states are in form of a self-sustained emission 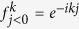, 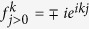 and a reflectionless absorption 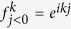, 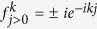 (see Method)^58^. The transfer matrix of the scattering centre is 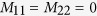, 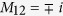, 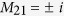 with matrix-element *M*_22_ vanishes^59^.

Now, we turn to discuss the rhombic ring structure under reflection 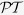 symmetry. The configuration is shown in Fig. 1d. Supplementary Fig. 2 schematically illustrates the pathways of photons. The connection sites are linked by two same pathways. In the presence of magnetic flux Φ, photons travelling from left lead to right lead in clockwise direction and counterclockwise direction acquire additional phases +Φ/2 and −Φ/2 in the two pathways (Supplementary Fig. 2c), respectively. The situation is unchanged for photons travelling inversely from right lead to left lead (Supplementary Fig. 2d). Equivalently, the upper and lower pathways are undistinguishable. Therefore, only relative phase difference Φ matters (affecting the transmission coefficient) and the transmission is directionless. The reflection and transmission coefficients are calculated as (see Methods)


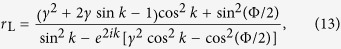



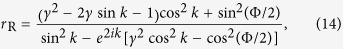



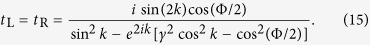


**Figure 4:**
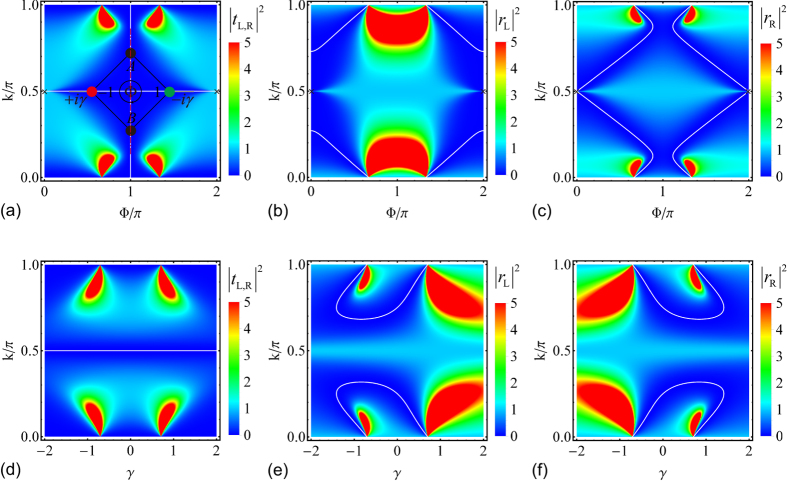
Reciprocal transmission and unidirectional reflection under reflection 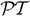 symmetry. (**a**–**c**) The reflection and transmission probabilities 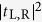, 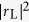, 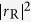 at *γ* = 1/2 as functions of Φ and *k*. (**d**–**f**) The reflection and transmission probabilities 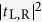, 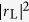, 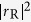 at Φ = *π*/2 as functions of *γ* and *k*. The insert in (**a**) schematically illustrates the reflection 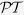-symmetric rhombic ring structure. The white curves are the reflection and transmission zeros. At *k* = *π*/2 and Φ = 0, 2*π*, the transmissions in (**a**) are 1, the reflections in (**b**,**c**) are 0 as marked by black crosses.

The reflection and transmission coefficients are functions of the magnetic flux Φ, gain/loss rate *γ*, and wave vector *k*. They satisfy 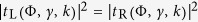 and 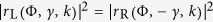. Figure 4 implies a reciprocal transmission (Fig. 4a,d) and unidirectional reflection (Fig. 4b,c,e,f). In this configuration, the scattering with both reflection and transmission being reciprocal happens in the absence of gain and loss (*γ* = 0), that is when the system is 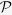-symmetric. In the presence of gain and loss (*γ* ≠ 0), the reflection probability is unidirectional, but the reflection 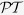 symmetry protects the reciprocal transmission. Due to the presence of gain and loss, the probability of the total reflection and transmission after scattering is not unitary, being balanced gain and loss rate dependent. The white curves in Fig. 4 show the reflection and transmission zeros. At *k* = *π*/2 and Φ = 0, 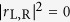, 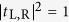. At the reflection zeros, 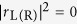, 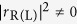, and 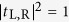, the system exhibits unidirectional reflectionless with reciprocal transmission.

**Figure 5:**
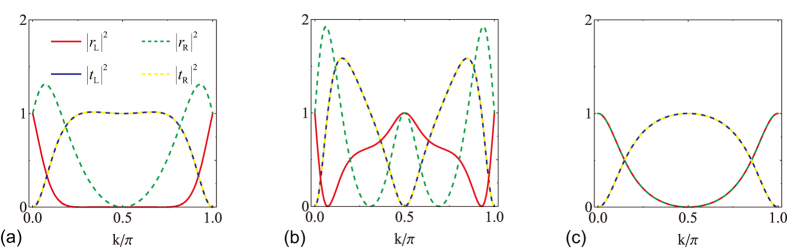
Symmetric transmission under reflection 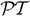 symmetry. (**a**) *γ* = 1/2, Φ = 0, (**b**) *γ* = 1/2, Φ = *π*/2, (**c**) *γ* = 0, Φ = 0.

In Fig. 5, we plot the reflection and transmission probabilities for a reflection 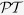-symmetric rhombic ring structure (Fig. 1d) at several set parameters. Figure 5a,b are for balanced gain and loss rate *γ* = 1/2 with two different magnetic flux Φ = 0 and *π*/2, respectively. The reciprocal transmission and unidirectional reflection are clearly seen. In Fig. 5a, the unidirectional reflectionless happens at *k* ≈ 0.27*π*, 0.73*π*, 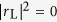, 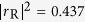, 
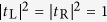
. In Fig. 5b, the unidirectional reflectionless happens at *k* ≈ 0.072*π*, 0.928*π*, 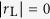, 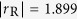, 
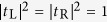
; or at *k* ≈ 0.310*π*, 0.690*π*, 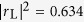, 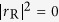, 
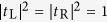
. In the absence of gain and loss *γ* = 0, the reflection and axial 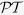-symmetric rhombic ring configurations reduce to an identical system. In Fig. 5c, we plots the reflection and transmission probabilities of a scattering centre in the absence of both gain and loss and magnetic flux, i.e., *γ* = 0, Φ = 0, we observe Hermitian scattering behavior of reciprocal reflection and transmission similar as *γ* = 0, Φ = *π*/2 shown in Fig. 3c. Notice that no spectral singularity emerges in the scattering of reflection 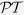-symmetric rhombic ring system. The system with Φ = 0 leads to input with wave vector *k* = *π*/2 both sides invisible that *r*_L_ = *r*_R_ = 0, *t*_L_ = *t*_R_ = 1 (black crosses in Fig. 4a–c); The system with Φ ≠ 0 leads to input with vector *k* = *π*/2 both sides opaque that *r*_L_ = *r*_R_ = 1, *t*_L_ = *t*_R_ = 0. For the input with wave vector *k* = *π*/2, the scattering behavior is very sensitive to the magnetic flux.

## Conclusion

We investigate the reciprocal and unidirectional scattering of 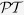-symmetric structures. We show an insightful understanding of the symmetric scattering behavior, that is associated with the type of 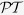 symmetry, defined as the 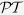 symmetry of the connection sites on the 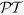-symmetric structures. We find that the axial (reflection) 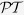 symmetry leads to reciprocal reflection (transmission). The transmission (reflection) is unidirectional affected by the magnetic flux and gain/loss, this is because the magnetic flux induced nonreciprocal phase and the gain/loss break the 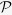 or 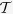 symmetry of the scattering centre. The results are further elucidated using a 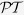-symmetric rhombic ring structure with enclosed effective magnetic flux describing by tight-binding model. The physical realization of such scattering centre is possible in optical systems such as coupled waveguides array and coupled resonators. Notice that our conclusions are also applicable to the system with nonreciprocal tunnelling being unequal tunnelling amplitude^60^. We believe our findings may shed light on coherent light transport and would be useful for applications of quantum devices with inherent symmetry, in particular, for novel unidirectional optical device designs that not limited to optical diodes using synthetic 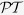-symmetric metamaterial.

## Methods

### Schrödinger equations

The input and output leads are described by two semi-infinite tight-binding chain. The left lead is 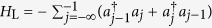, the right lead is 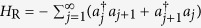, where 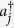 (*a*_*j*_) is the creation (annihilation) operator of the site *j*, the tunnelling between sites is uniform and set unity. The Hamiltonian of the scattering system is *H* = *H*_L_ + *H*_c_ + *H*_R_. The eigenstate of the scattering system is set 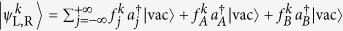.

For the axial 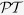-symmetric configuration shown in Fig. 1c, the Hamiltonian of the scattering system is 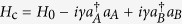. The Schrödinger equations 
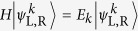
 on the scattering centre yield four independent equations


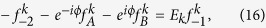



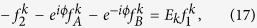



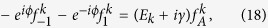



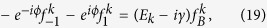


For the reflection 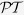-symmetric configuration shown in Fig. 1d, the Hamiltonian of the scattering centre is 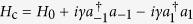. Correspondingly, four independent equations from the Schrödinger equations 
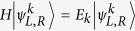
 on the scattering centre are in form of


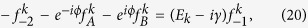



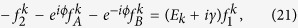



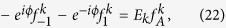



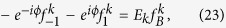


where *ϕ* = Φ/4. The Schrödinger equations on the leads give the energy *E*_*k*_ = −2cos*k* for the input with wave vector *k*. Notice that *k* = *π*/2 in the reflection 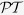-symmetric rhombic ring with Φ = 2*nπ* 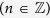 results in 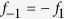 and the transmissions are 1. Otherwise, *k* = *π*/2 in system with 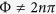 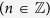 leads to *f*_−1_ = *f*_1_ = 0 and the transmissions are 0.

### Reflection and transmission coefficients

To calculate the reflection and transmission coefficients, we set the left side input wave functions equation (1) as


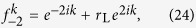



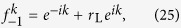



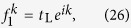



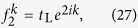


and the right side input wave functions equation (2) as


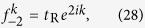



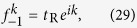



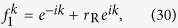



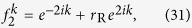


Substituting 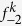, 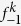, 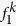, 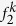 of equations (24, 25, 26, 27, 28, 29, 30, 31) into equations (16, 17, 18, 19) and eliminating 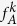, 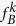, we get equations of *r*_L_, *t*_L_, *r*_R_, *t*_R_ for the axial 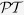-symmetric rhombic ring configuration. Through directly algebraic calculation and simplification, we obtain the reflection and transmission coefficients *r*_L_, *t*_L_, *r*_R_, *t*_R_ as functions of *k*, Φ, *γ* given in equations (10, 11, 12). Using the same procedure, we get the reflection and transmission coefficients for the reflection 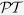-symmetric rhombic ring configuration. After substituting 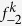, 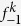, 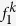, 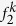 of equations (24, 25, 26, 27, 28, 29, 30, 31) into equations (20, 21, 22, 23) and eliminating 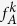, 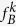, we get equations of *r*_L_, *t*_L_, *r*_R_, *t*_R_. Through directly algebraic calculation and simplification, we obtain the reflection and transmission coefficients *r*_L_, *t*_L_, *r*_R_, *t*_R_ as functions of *k*, Φ, *γ* given in equations (13, 14, 15).

### Scattering states at the spectral singularities

The scattering coefficients diverge at the spectral singularities, to calculate the scattering states, we have the wave functions of equation (1) replaced by 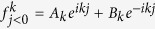, 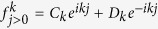. Substituting 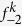, 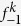, 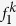, 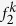 into equations (16, 17, 18, 19) of the axial 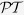-symmetric rhombic ring configuration, we obtain the coefficients satisfying 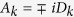, 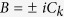 at the spectral singularities that i) *k* = *π*/2, 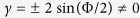; and ii) Φ = *π*, 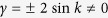. These indicate the scattering states are a self-sustained emission and a reflectionless absorption.

## Additional Information

**How to cite this article**: Jin, L. *et al.* Reciprocal and unidirectional scattering of parity-time symmetric structures. *Sci. Rep.* **6**, 20976; doi: 10.1038/srep20976 (2016).

## Supplementary Material

Supplementary Information
