# Supplementary material for: Reciprocal and unidirectional scattering of parity-time symmetric structures
Source: Sci Rep. 2016 Feb 15;6:20976. doi: 10.1038/srep20976 (PMC4753482; doi:10.1038/srep20976)
Supplement: Supplementary Information [file srep20976-s1.pdf]

Reciprocal and unidirectional scattering of $\mathcal{PT}$-symmetric structures

L. Jin,[1,*] X. Z. Zhang,[2] G. Zhang,[1] and Z. Song[1]

[1]*School of Physics, Nankai University, Tianjin 300071, P. R. China*

[2]*College of Physics and Materials Science,*
Tianjin Normal University, Tianjin 300387, P. R. China

[*] jinliang@nankai.edu.cn

SUPPLEMENTARY INFORMATION

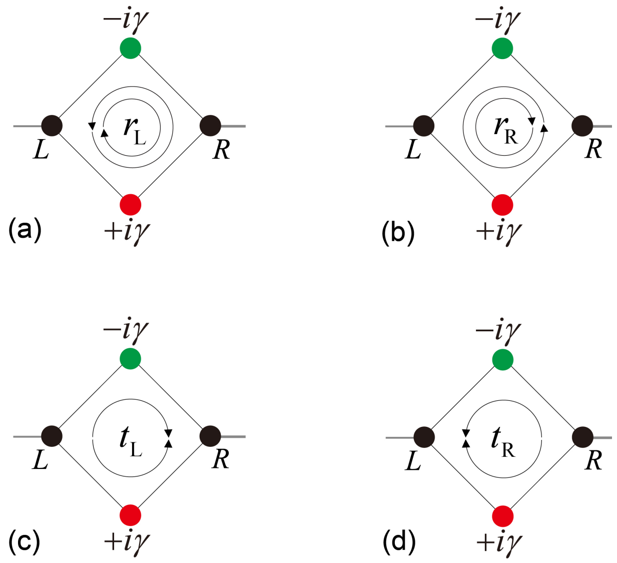

Supplementary Figure 1. **Photon pathways of axial $\mathcal{PT}$-symmetric rhombic ring.** The arrows show the pathways in clockwise/counterclockwise direction for (a) r_L, (b) r_R, (c) t_L, (d) t_R.

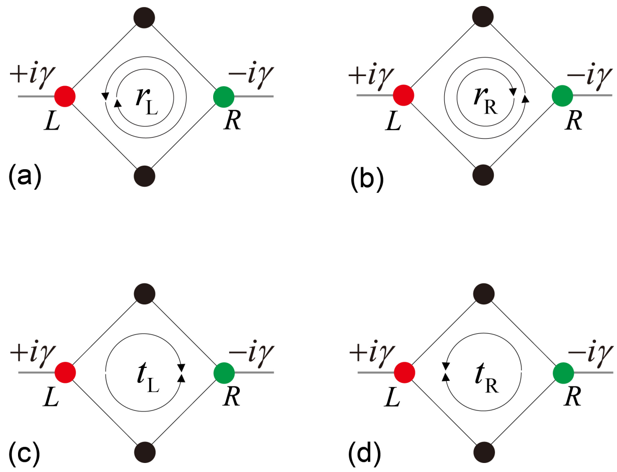

Supplementary Figure 2. **Photons pathways of reflection $\mathcal{PT}$-symmetric rhombic ring.** The arrows show the pathways in clockwise/counterclockwise direction for (a) r_L, (b) r_R, (c) t_L, (d) t_R.

In the Supplementary Information, we show the reciprocal reflection (transmission) for axial (reflection) $\mathcal{PT}$ symmetry in details. The input and output leads are described by two uniform semi-infinite tight-binding chain connected to the scattering centre. The wave functions for the left and right lead input with wave vector k are ψ_{L}^{k} and ψ_{R}^{k}, in form of

$$\psi_{\mathrm{L}}^{k}(j)=\begin{cases} e^{ikj}+r_{\mathrm{L}}e^{-ikj}, j<0 \\ t_{\mathrm{L}}e^{ikj}, j>0 \end{cases}, \tag{1}$$

$$\psi_{\mathrm{R}}^{k}(j)=\begin{cases} t_{\mathrm{R}}e^{-ikj}, j<0 \\ e^{-ikj}+r_{\mathrm{R}}e^{ikj}, j>0 \end{cases}. \tag{2}$$

where $r_{\mathrm{L,R}}$ and $t_{\mathrm{L,R}}$ represent corresponding reflection and transmission coefficients of wave with vector k.

SUPPLEMENTARY NOTE 1. RECIPROCAL REFLECTION UNDER AXIAL $\mathcal{PT}$ SYMMETRY

In this situation (Fig. 1a), the whole scattering system is axial $\mathcal{PT}$-symmetric with respect to the leads, where $(\mathcal{PT})\,H_{\mathrm{L}}\,(\mathcal{PT})^{-1}=H_{\mathrm{L}}$, $(\mathcal{PT})\,H_{\mathrm{R}}\,(\mathcal{PT})^{-1}=H_{\mathrm{R}}$, and $(\mathcal{PT})\,H_{\mathrm{c}}\,(\mathcal{PT})^{-1}$ $=H_{\mathrm{c}}$. The $\mathcal{PT}$ symmetry of the scattering system results in symmetric relations on its wave functions. In order to reveal the symmetry properties hidden in the scattering wave functions, we act the $\mathcal{PT}$ operator on the wave function of left lead input $\psi_{\mathrm{L}}^{k}(j)$, and get

$$\mathcal{PT}\psi_{\mathrm{L}}^{k}(j)=\begin{cases} e^{-ikj}+r_{\mathrm{L}}^{*}e^{ikj}, j<0 \\ t_{\mathrm{L}}^{*}e^{-ikj}, j>0 \end{cases}, \tag{3}$$

we act $\mathcal{PT}$ operator on the wave function of right lead input $\psi_{\mathrm{R}}^{k}(j)$, and get

$$\mathcal{PT}\psi_{\mathrm{R}}^{k}(j)=\begin{cases} t_{\mathrm{R}}^{*}e^{ikj}, j<0 \\ e^{ikj}+r_{\mathrm{R}}^{*}e^{-ikj}, j>0 \end{cases}. \tag{4}$$

The real energy scattering state is $\mathcal{PT}$-symmetric due to the $\mathcal{PT}$ symmetry of the whole scattering system. Moreover, the two series of wave functions (the eigenstates before and after acting the $\mathcal{PT}$ operator) are both eigenstates of the system with eigenvalue $E_k=-2\cos k$. Therefore, they must be in accords with each other, i.e., we can use $\psi_{\mathrm{L}}^{k}(j)$ and $\psi_{\mathrm{R}}^{k}(j)$ to compose $\mathcal{PT}\psi_{\mathrm{L}}^{k}(j)$ and $\mathcal{PT}\psi_{\mathrm{R}}^{k}(j)$, because the left side input and right side input scattering states are degenerate.

Composing $\mathcal{PT}\psi_{\rm L}^{k}(j)$ via $\psi_{\rm L}^{k}(j)$ and $\psi_{\rm R}^{k}(j)$ of equations (1, 2) by eliminating e^{ikj} in $j>0$ region and comparing the coeffients of $e^{\pm ikj}$ in the result with $\mathcal{PT}\psi_{\rm L}^{k}(j)$, we obtain

$$t_{\rm L}^{*}\left(t_{\rm L}t_{\rm R}-r_{\rm L}r_{\rm R}\right)=t_{\rm L}, \tag{5}$$

$$-r_{\rm R}t_{\rm L}^{*}=r_{\rm L}^{*}t_{\rm L}, \tag{6}$$

Composing $\mathcal{PT}\psi_{\rm R}^{k}(j)$ via $\psi_{\rm L}^{k}(j)$ and $\psi_{\rm R}^{k}(j)$ of equations (1, 2) by eliminating e^{-ikj} in $j<0$ region and comparing the coeffients of $e^{\pm ikj}$ in the result with $\mathcal{PT}\psi_{\rm R}^{k}(j)$, we obtain

$$t_{\rm R}^{*}\left(t_{\rm L}t_{\rm R}-r_{\rm L}r_{\rm R}\right)=t_{\rm R}, \tag{7}$$

$$-r_{\rm L}t_{\rm R}^{*}=r_{\rm R}^{*}t_{\rm R}, \tag{8}$$

we simplify the relations in equations (5, 6), and get

$$|r_{\rm L}|^{2}+t_{\rm L}^{*}t_{\rm R}=1, \tag{9}$$

$$r_{\rm L}^{*}t_{\rm L}+r_{\rm R}t_{\rm L}^{*}=0, \tag{10}$$

we simplify the relations in equations (7, 8), and get

$$|r_{\rm R}|^{2}+t_{\rm L}t_{\rm R}^{*}=1, \tag{11}$$

$$r_{\rm R}^{*}t_{\rm R}+r_{\rm L}t_{\rm R}^{*}=0, \tag{12}$$

From equations (9, 11), we obtain the reciprocal reflection for axial $\mathcal{PT}$ symmetry.

$$|r_{\rm L}|^{2}=|r_{\rm R}|^{2}. \tag{13}$$

Moreover, considering the wave vector k and $-k$, we obtain $r_{\rm L}(-k)=r_{\rm L}^{*}(k)$, $t_{\rm L}(-k)=t_{\rm L}^{*}(k)$ by comparing $\mathcal{PT}\psi_{\rm L}^{k}(j)$ and $\psi_{\rm L}^{-k}(j)$; and we obtain $r_{\rm R}(-k)=r_{\rm R}^{*}(k)$, $t_{\rm R}(-k)=t_{\rm R}^{*}(k)$ by comparing $\mathcal{PT}\psi_{\rm R}^{k}(j)$ and $\psi_{\rm R}^{-k}(j)$.

SUPPLEMENTARY NOTE 2. RECIPROCAL TRANSMISSION UNDER REFLECTION $\mathcal{PT}$ SYMMETRY

In this situation (Fig. 1b), the whole scattering system is reflection $\mathcal{PT}$-symmetric with respect to the leads, where $\left(\mathcal{PT}\right)H_{\rm L}\left(\mathcal{PT}\right)^{-1}=H_{\rm R}$, $\left(\mathcal{PT}\right)H_{\rm R}\left(\mathcal{PT}\right)^{-1}=H_{\rm L}$, and $\left(\mathcal{PT}\right)H_{\rm c}\left(\mathcal{PT}\right)^{-1}=H_{\rm c}$. The $\mathcal{PT}$ symmetry of the scattering system results in symmetric relations on its wave functions. In order to reveal the symmetry properties hidden in the

scattering wave function, we act the $\mathcal{PT}$ operator on the wave function of the left lead input $\psi_{\rm L}^{k}(j)$ similarly as the previous analysis, and get

$$\mathcal{PT}\psi_{\rm L}^{k}(j)=\begin{cases} t_{\rm L}^{*}e^{ikj}, j<0 \\ e^{ikj}+r_{\rm L}^{*}e^{-ikj}, j>0 \end{cases}, \tag{14}$$

we act the $\mathcal{PT}$ operator on the wave function of right lead input $\psi_{\rm R}^{k}(j)$, and get

$$\mathcal{PT}\psi_{\rm R}^{k}(j)=\begin{cases} e^{-ikj}+r_{\rm R}^{*}e^{ikj}, j<0 \\ t_{\rm R}^{*}e^{-ikj}, j>0 \end{cases}. \tag{15}$$

We take $\psi_{\rm L}^{k}(j)$ and $\psi_{\rm R}^{k}(j)$ to compose $\mathcal{PT}\psi_{\rm L}^{k}(j)$ and $\mathcal{PT}\psi_{\rm R}^{k}(j)$. Composing $\psi_{\rm L}^{k}(j)$ and $\psi_{\rm R}^{k}(j)$ of equations (1, 2) in $j<0$ region by eliminating e^{-ikj} and comparing the coeffients of $e^{\pm ikj}$ in the result with $\mathcal{PT}\psi_{\rm L}^{k}(j)$, we obtain

$$t_{\rm L}^{*}\left(t_{\rm L}t_{\rm R}-r_{\rm L}r_{\rm R}\right)=t_{\rm R}, \tag{16}$$

$$-t_{\rm L}^{*}r_{\rm L}=r_{\rm L}^{*}t_{\rm R}, \tag{17}$$

Composing $\psi_{\rm L}^{k}(j)$ and $\psi_{\rm R}^{k}(j)$ of equations (1, 2) in $j>0$ region by eliminating e^{ikj} and comparing the coeffients of $e^{\pm ikj}$ in the result with $\mathcal{PT}\psi_{\rm R}^{k}(j)$, we obtain

$$t_{\rm R}^{*}\left(t_{\rm L}t_{\rm R}-r_{\rm L}r_{\rm R}\right)=t_{\rm L}, \tag{18}$$

$$-r_{\rm R}t_{\rm R}^{*}=r_{\rm R}^{*}t_{\rm L}, \tag{19}$$

we simplify the relations in equations (16, 17), and get

$$|t_{\rm L}|^{2}+r_{\rm L}^{*}r_{\rm R}=1, \tag{20}$$

$$r_{\rm L}t_{\rm L}^{*}+r_{\rm L}^{*}t_{\rm R}=0, \tag{21}$$

we simplify the relations in equations (18, 19), and get

$$|t_{\rm R}|^{2}+r_{\rm L}r_{\rm R}^{*}=1, \tag{22}$$

$$r_{\rm R}^{*}t_{\rm L}+r_{\rm R}t_{\rm R}^{*}=0, \tag{23}$$

From equations (20, 22), we obtain the reciprocal transmission for reflection $\mathcal{PT}$ symmetry.

$$|t_{\rm L}|^{2}=|t_{\rm R}|^{2}. \tag{24}$$

Moreover, considering the wave vector k and $-k$, we obtain $r_{\rm L}(-k)=r_{\rm R}^{*}(k)$, $t_{\rm L}(-k)=t_{\rm R}^{*}(k)$ by comparing $\mathcal{PT}\psi_{\rm R}^{k}(j)$ and $\psi_{\rm L}^{-k}(j)$; and we obtain $r_{\rm R}(-k)=r_{\rm L}^{*}(k)$, $t_{\rm R}(-k)=t_{\rm L}^{*}(k)$ by comparing $\mathcal{PT}\psi_{\rm L}^{k}(j)$ and $\psi_{\rm R}^{-k}(j)$.
